# A Multicenter, Prospective Real‐World Study of Simethicone–Activated Charcoal in Patients With Persistent Dyspeptic Symptoms Despite Acid Suppression (SIMAC Study)

**DOI:** 10.1002/jgh3.70475

**Published:** 2026-10-01

**Authors:** Ashish Kumar, D. V. Sreenivas, Sambit Kumar Behera, Guduru Raghavendra Srinivas Rao, G. S. Sameer Kumar, Jaya Ghosh Chatterjee, Nalini Kant Choudhary, Ravi Shankar Bagepally, Shekhar Puri, S. M. A. Rizvi, Rahul Pathak, Shruti Pal

**Affiliations:** ^1^ Department of Gastroenterology & Hepatology Sir Ganga Ram Hospital Delhi India; ^2^ Department of Gastroenterology Mythri Hospital Hyderabad India; ^3^ Department of Gastroenterology MKCG Medical College Berhampur Odisha India; ^4^ Department of Gastroenterology Yashoda Hospital Secunderabad Telangana India; ^5^ Department of Medical Gastroenterology Gleneagles Hospital Hyderabad India; ^6^ Department of Gastroenterology Peerless Hospital Kolkata West Bengal India; ^7^ Department of Surgery Bokaro Surgical Clinic Bokaro India; ^8^ Department of Medical Gastroenterology Yashoda Hospitals Secunderabad India; ^9^ Department of Gastroenterology and Hepatology Samarpan Gastro & Liver Clinic Varanasi India; ^10^ Department of General Medicine Dr. KNS Memorial Hospital (Mayo Medical Centre) Lucknow India; ^11^ Medical Affairs, Indchemie Health Specialities Pvt. Ltd. India

**Keywords:** activated charcoal, bloating, functional dyspepsia, gas‐related symptoms, proton pump inhibitor, real‐world study, simethicone

## Abstract

**Background:**

Persistent gas‐predominant dyspeptic symptoms affect many patients despite adequate proton pump inhibitor (PPI) therapy, reflecting mechanisms beyond acid secretion. We evaluated the real‐world effectiveness and safety of simethicone (80 mg) plus activated charcoal (250 mg) in adults with persistent symptoms despite prior PPI therapy.

**Methods:**

In this prospective, multicenter observational study across nine Indian sites, adults with persistent gas‐related symptoms despite ≥ 2 weeks of standard‐dose PPI therapy received the combination thrice daily for 28 days. Severity across 11 gastrointestinal domains was assessed using a modified Visual Analog Severity scale at baseline, Day‐14, and 28. The primary outcome was change in symptom severity from baseline to Day 28, analyzed using Friedman testing with Bonferroni‐adjusted Wilcoxon post hoc comparisons. A global symptom score (mean of all 11 domains) was used for exploratory subgroup analyses; secondary outcomes included adherence and safety.

**Results:**

All 183 participants completed follow‐up, with significant reductions across all 11 symptoms (all *p* < 0.001). Belching decreased from 46.3 ± 26.4 to 10.9 ± 19.5, epigastric burning from 48.1 ± 27.3 to 10.7 ± 17.9, and bloating from 44.2 ± 29.1 to 12.9 ± 18.6. Improvements were evident by Day‐14 and sustained to Day‐28. The median global symptom score reduction was 25.0 units (95% CI: 22.7–27.3), with consistent improvement across demographic and clinical subgroups. Full adherence was 87.1% at Day 14 and 83.6% at Day 28, with no serious clinical events.

**Conclusion:**

In patients with persistent gas‐predominant dyspepsia despite PPI therapy, simethicone–activated charcoal was associated with significant short‐term symptomatic improvement. Controlled trials are warranted to confirm treatment effect.

## 1 Introduction

Chronic dyspepsia is a heterogeneous clinical syndrome characterized by persistent or recurrent upper gastrointestinal symptoms that significantly impair quality of life [1, 2, 3, 4, 5]. Functional dyspepsia (FD) is diagnosed when symptoms persist for at least 3 months, with onset at least 6 months prior to diagnosis, in the absence of any structural or biochemical abnormality that can explain the symptoms [6, 7]. It is a highly prevalent condition, with a pooled global prevalence of 8.4% based on Rome criteria [8]. In India, the burden is even more pronounced; the Power 1.0 study demonstrated that nearly 78.5% of patients presenting with dyspepsia had functional disease [9]. FD is conventionally classified into epigastric pain syndrome and postprandial distress syndrome [7], though considerable overlap with gastroesophageal reflux disease and other functional gastrointestinal disorders is well recognized [10, 11, 12].

Acid suppression with proton pump inhibitors (PPIs) remains the most commonly used first‐line therapy for dyspepsia [13], with potassium‐competitive acid blockers such as vonoprazan emerging as newer alternatives [14]. However, a substantial proportion of patients continue to experience incomplete symptom relief despite adequate acid suppression [15, 16]. In particular, gas‐predominant symptoms—bloating, belching, abdominal distension, excessive flatulence, and difficulty in gas evacuation—often persist, as these are primarily driven by abnormalities in luminal gas handling, visceral hypersensitivity, and mechanical distension rather than by excess gastric acid. Further escalation of acid‐suppressive therapy is unlikely to benefit this subgroup, and prolonged PPI use itself has been associated with bloating, flatulence, and small intestinal bacterial overgrowth through alterations in gastric acidity and gut microbiota composition [17]. These considerations highlight the need for complementary therapeutic approaches targeting intraluminal gas.

Simethicone is a non‐systemic anti‐foaming agent that reduces the surface tension of gas bubbles, facilitating their coalescence and expulsion [18, 19, 20]. Activated charcoal complements this action by adsorbing excess intestinal gas and other luminal substances contributing to gaseous distension [21, 22]. The combination therefore addresses the luminal and mechanical components of dyspeptic symptoms inadequately targeted by acid suppression alone. The fixed‐dose combination of simethicone (80 mg) and activated charcoal (250 mg) was approved by the Drug Controller General of India (DCGI) in 2015 for the symptomatic management of gas‐related gastrointestinal discomfort.

Despite widespread clinical use of this combination in India, robust real‐world evidence regarding its effectiveness, safety, and acceptability remains limited. The present multicenter, prospective, observational study was undertaken to evaluate the real‐world effectiveness and safety of this fixed‐dose combination in Indian adults with persistent gas‐predominant dyspeptic symptoms despite standard acid‐suppressive therapy.

## 2 Patients and Methods

### 2.1 Study Design and Population

This was a prospective, multicenter, single‐arm, observational real‐world study conducted across nine clinical sites in India over a 14‐month period (20 June 2024 to 31 August 2025). The study was designed to evaluate a complementary, gas‐targeted therapeutic approach in routine clinical practice rather than further escalation of acid‐suppressive therapy.

Adult patients (≥ 18 years) with dyspepsia consistent with functional dyspepsia in routine clinical practice, who continued to report predominant gas‐related symptoms such as bloating, belching, abdominal distension, or excessive flatulence despite at least 2 weeks of standard‐dose proton pump inhibitor (PPI) therapy, were eligible for enrollment.

The diagnosis of functional dyspepsia was established by treating physicians through routine clinical evaluation, generally involving symptom assessment consistent with Rome IV criteria and exclusion of organic pathology when clinically indicated. No protocol‐mandated diagnostic procedures were required.

Similarly, inadequate response to prior PPI therapy was determined by physician assessment rather than by a formal validated symptom scale. Compliance with the preceding two‐week PPI course was confirmed at enrollment through physician verification, and only patients who continued to report persistent dyspeptic symptoms were included.

Eligible participants were prescribed a fixed‐dose combination of simethicone (80 mg) and activated charcoal (250 mg) (Indchemie Health Specialities Pvt. Ltd., Mumbai, India) three times daily, 1 h after meals, for 28 days as part of routine care. Activated charcoal refers to pharmaceutical‐grade activated carbon prepared by controlled carbonization and activation to achieve high surface area for intraluminal gas adsorption, in accordance with pharmacopoeial standards. The therapy was prescribed either as an add‐on to ongoing acid‐suppressive treatment (*n* = 142; 89.3%) or as a replacement, defined as discontinuation of PPI and substitution with the study combination as sole therapy (*n* = 17; 10.7%), at the discretion of the treating physician; treatment strategy data were unavailable for 24 participants. Follow‐up assessments were conducted either in person or telephonically as per routine clinical practice.

Key exclusion criteria included known hypersensitivity to simethicone or activated charcoal, history of gastrointestinal obstruction, perforation, or active gastrointestinal bleeding, and concurrent use of antibiotics commonly prescribed for flatulence, such as rifaximin, ofloxacin, or ornidazole.

### 2.2 Data Collection and Outcome Measures

A structured case record form was used to collect demographic details, comorbidities, medication history, and relevant clinical information as part of routine clinical documentation. Comorbidities including diabetes mellitus, hypertension, obesity, constipation, and irritable bowel syndrome were recorded based on the treating physician's clinical assessment; formal diagnostic criteria (such as Rome IV for IBS) were not mandated by the protocol.

The primary outcome was change in overall symptom intensity from baseline to Day 28. Secondary outcomes included treatment adherence, clinician‐rated global satisfaction, likelihood of recommendation, and assessment of safety and tolerability.

Symptom severity for key gas‐related complaints was assessed across 11 gastrointestinal domains. The symptom domains were selected based on the Intestinal Gas Questionnaire (IGQ) framework [23, 24], with the addition of epigastric burning [25] and gas incontinence; however, the modified instrument as used in this study has not been independently validated and no claim of validity is made based on the original IGQ. The predefined symptoms included stomach rumbling, difficulty in gas evacuation, gas incontinence, excessive flatus volume, malodorous flatus, excessive frequency of flatulence, belching/burping, epigastric burning or discomfort, abdominal distension, bloating, and bad breath. Each symptom was graded on a simplified five‐point ordinal severity scale anchored at 0 (no symptoms), 25 (mild), 50 (moderate), 75 (severe), and 100 (very severe). For descriptive purposes, scores are reported as means with standard deviations to convey overall trends, while inferential analyses employed non‐parametric methods appropriate for ordinal data. Assessments were performed at baseline (Day 0), Day 14, and Day 28. A global symptom score was derived for each participant as the arithmetic mean of all 11 individual domain scores at each time point; this is an unvalidated pragmatic composite used solely for exploratory subgroup comparisons and should not be interpreted as a psychometrically validated summary instrument. Change in global symptom score from baseline to Day 28 was used for exploratory subgroup analyses. Medication adherence was assessed using a four‐level self‐reported compliance scale as recorded in the case record form.

### 2.3 Ethical Approval and Informed Consent

The study was approved by the Royal Pune Independent Ethics Committee, Pune (Registration No. ECR/45/Indt/MH/2013/RR‐19). The study was conducted in accordance with the principles of the Declaration of Helsinki and the International Council for Harmonisation Good Clinical Practice (ICH‐GCP) guidelines, along with applicable Indian regulatory and ethical standards.

Written informed consent was obtained from all participants prior to enrollment. Patient confidentiality was maintained through anonymization and the use of unique identifiers, and collected data were used solely for the purposes of this study.

### 2.4 Statistical Analysis

Data were analyzed in aggregated, de‐identified form using Python software. Continuous variables were expressed as means with standard deviations, and categorical variables were summarized as frequencies and percentages.

Changes in symptom severity across the three time points (baseline, Day 14, and Day 28) were analyzed using the Friedman repeated‐measures test for each of the 11 symptom domains. Where the Friedman test indicated a significant overall difference, Bonferroni‐adjusted Wilcoxon signed‐rank post hoc comparisons were performed for each pairwise interval (baseline vs. Day 14, baseline vs. Day 28, and Day 14 vs. Day 28). Categorical comparisons were performed using the Chi‐square test where applicable.

For exploratory subgroup analyses, between‐group differences in median global symptom score change were compared using the Mann–Whitney *U* test. Subgroups were defined by age (≤ 45 vs. > 45 years), sex, obesity status (BMI ≥ 25 vs. < 25 kg/m^2^), diabetes, hypertension, constipation, and irritable bowel syndrome. Given the number of subgroup comparisons, results were interpreted with consideration for multiple testing.

No formal sample size calculation was performed; enrollment was based on consecutive eligible patients across participating sites during the study period. Post hoc analysis confirmed that the achieved sample of 183 participants provided > 99% power to detect a within‐subject change of 10 points on the 0–100 severity scale (the lower bound of accepted minimal clinically important difference thresholds for analogous VAS instruments) at a two‐sided alpha of 0.05, based on the observed standard deviations of change scores. Clinician‐rated treatment satisfaction was assessed at Day 28 using a five‐point Likert scale for management of dyspepsia and chronic flatulence, and likelihood of recommendation was assessed on a 0–10 numeric rating scale anchored at 0 (“not at all likely’”) and 10 (“extremely likely” to recommend the combination to a friend or relative). A comparison of symptomatic improvement between add‐on and replacement therapy strategies was performed using the Mann–Whitney *U* test. Temporal response patterns were characterized using mutually exclusive categories: early responders (any reduction in severity score from baseline by Day 14, irrespective of further change thereafter), late responders (no reduction from baseline at Day 14 but improved by Day 28), and non‐responders (no reduction from baseline at either time point). Results of these analyses are reported in the Suupporting Information: Appendix. A *p*‐value < 0.05 was considered statistically significant. Microsoft Excel was used for data tabulation and graphical presentation.

## 3 Results

### 3.1 Study Population

The study flow is summarized in Figure 1. A total of 183 participants were enrolled and received simethicone–activated charcoal therapy. All participants completed follow‐up at Day 14 and Day 28, and all were included in the final analysis. The absence of attrition likely reflects the short study duration, non‐invasive nature of the intervention, and the option for telephonic follow‐up.

**FIGURE 1:**
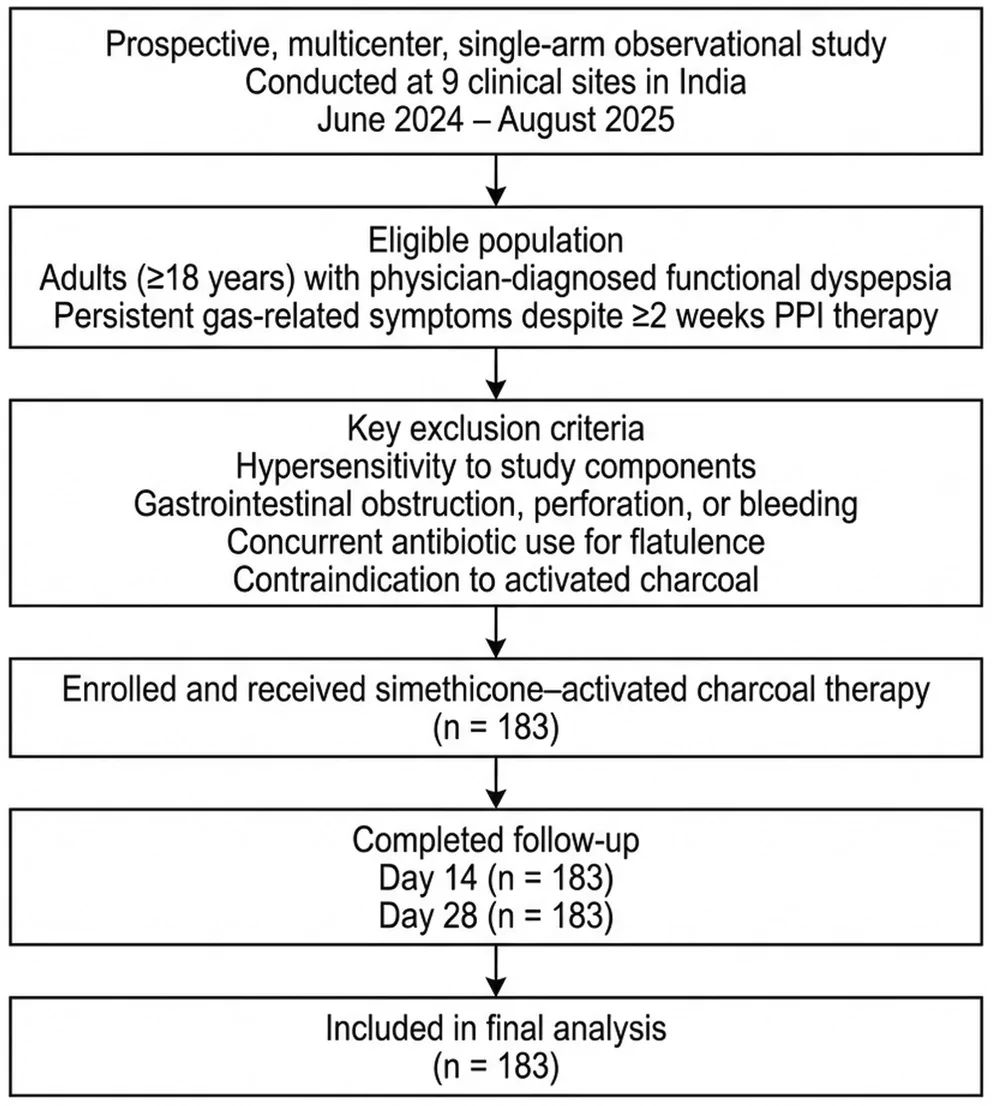
Study enrollment and follow‐up flow.

### 3.2 Baseline Characteristics

The baseline characteristics of the study cohort are summarized in Table 1. The mean age was 47.7 ± 14.7 years, and 63% of participants were male. The mean body mass index (BMI) was 26.4 ± 5.0 kg/m^2^, with the majority classified as overweight or obese according to Indian consensus criteria.

**TABLE 1:** Baseline characteristics of study participants.

Variable	Value
Total participants	183
Age, years (mean ± SD)	47.7 ± 14.7
Male sex, *n* (%)	115 (62.8)
BMI, kg/m^2^ (mean ± SD)	26.4 ± 5.0
Obesity (BMI ≥ 25 kg/m^2^), *n* (%)	116 (63.4)
Key Comorbidities, *n* (%)	
Diabetes mellitus	52 (40.0)
Hypertension	43 (33.1)
Irritable bowel syndrome	36 (27.7)
Chronic constipation	36 (27.7)
Coronary artery disease	6 (3.3)
Social habits, *n* (%)	
Alcohol use	39 (24.1)
Smoking	34 (20.6)
Prior acid‐suppressive therapy, *n* (%)	
Pantoprazole	59 (35.8)
Omeprazole	55 (33.3)
Esomeprazole	46 (27.9)
Rabeprazole	34 (20.6)
Dexlansoprazole	10 (6.1)
Famotidine	6 (3.6)
Ranitidine	3 (1.8)

Metabolic and functional gastrointestinal comorbidities were common. Diabetes mellitus and hypertension were the most frequently reported systemic conditions, while irritable bowel syndrome and constipation were the predominant functional gastrointestinal comorbidities. Approximately one‐quarter of participants reported alcohol consumption, and one‐fifth had a history of smoking.

Prior to enrollment, proton pump inhibitors were the most commonly used acid‐suppressive agents, particularly pantoprazole, omeprazole, and esomeprazole. Some patients had received more than one PPI sequentially prior to enrollment. A subset of participants was also receiving prokinetic therapy. All patients continued to report persistent gas‐related symptoms despite prior treatment.

### 3.3 Effectiveness of Simethicone–Activated Charcoal Combination

Changes in symptom severity distributions over time are illustrated in Figure 2. Across all 11 evaluated symptoms, treatment was associated with a progressive shift toward lower severity categories from baseline to Day 14 and further to Day 28. The proportion of participants reporting no or mild symptoms increased consistently across all domains.

**FIGURE 2:**
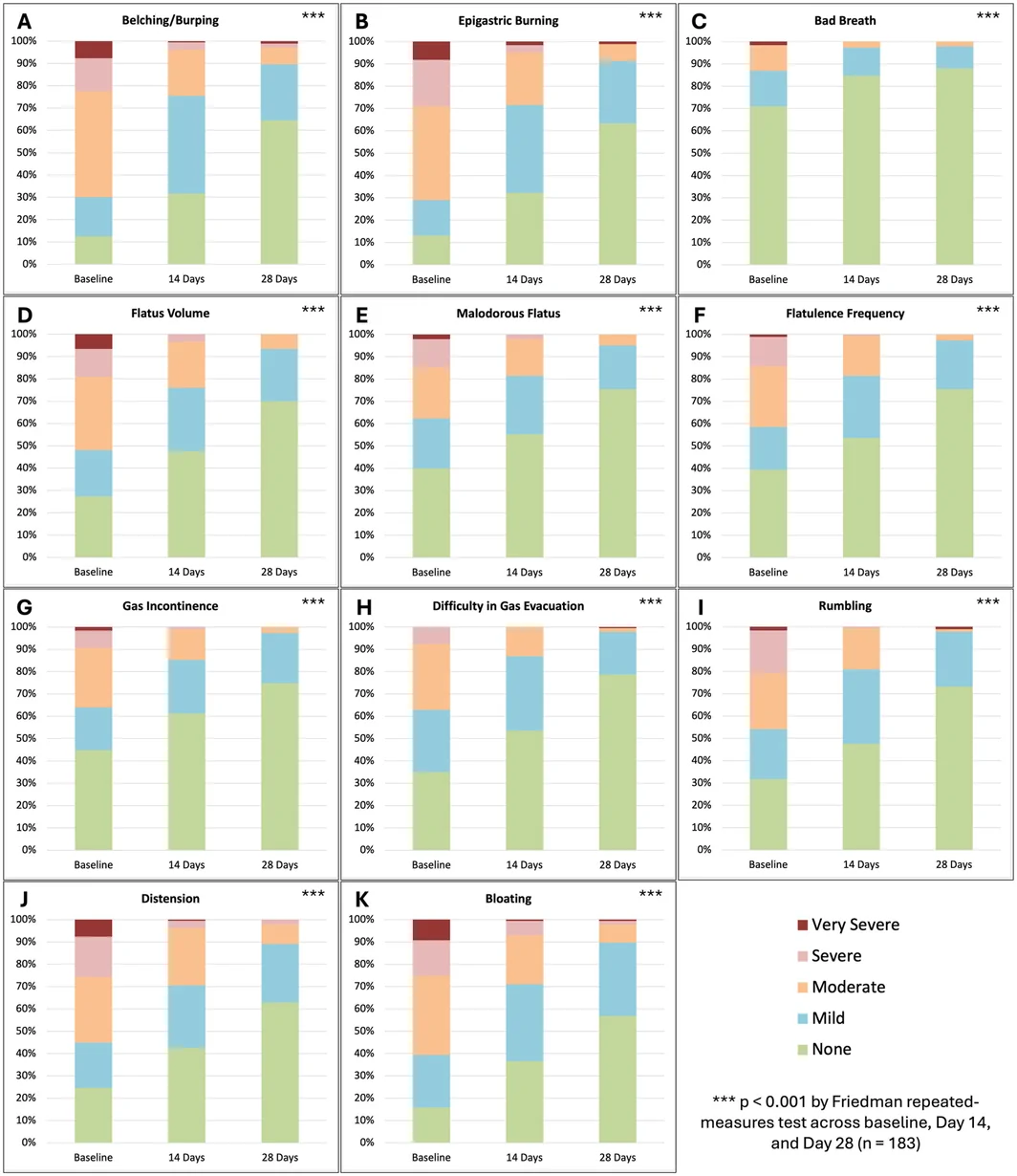
Distribution of symptom severity categories (VAS 0–100) at baseline, Day 14, and Day 28 for 11 gastrointestinal symptoms. Stacked bars demonstrate a progressive shift toward lower severity categories over time. ***p < 0.001 by Friedman repeated‐measures test across all time points (*n* = 183).

#### 3.3.1 Upper Gastrointestinal Symptoms

Belching/burping, epigastric burning, and bad breath demonstrated marked reductions in mean VAS scores over the 28‐day period. Mean belching/burping scores decreased from 46.3 ± 26.4 at baseline to 23.9 ± 20.9 at Day 14 and 10.9 ± 19.5 at Day 28. Epigastric burning declined from 48.1 ± 27.3 to 24.8 ± 22.2 and 10.7 ± 17.9, respectively. Bad breath severity reduced from 10.8 ± 20.0 at baseline to 4.1 ± 10.5 at Day 14 and 2.4 ± 8.7 at Day 28 (Figure 3). Friedman repeated‐measures testing demonstrated statistically significant differences across time points for each symptom (*p* < 0.001), with Bonferroni‐adjusted Wilcoxon post hoc analysis confirming significant reductions between baseline and Day 14, baseline and Day 28, and Day 14 and Day 28.

**FIGURE 3:**
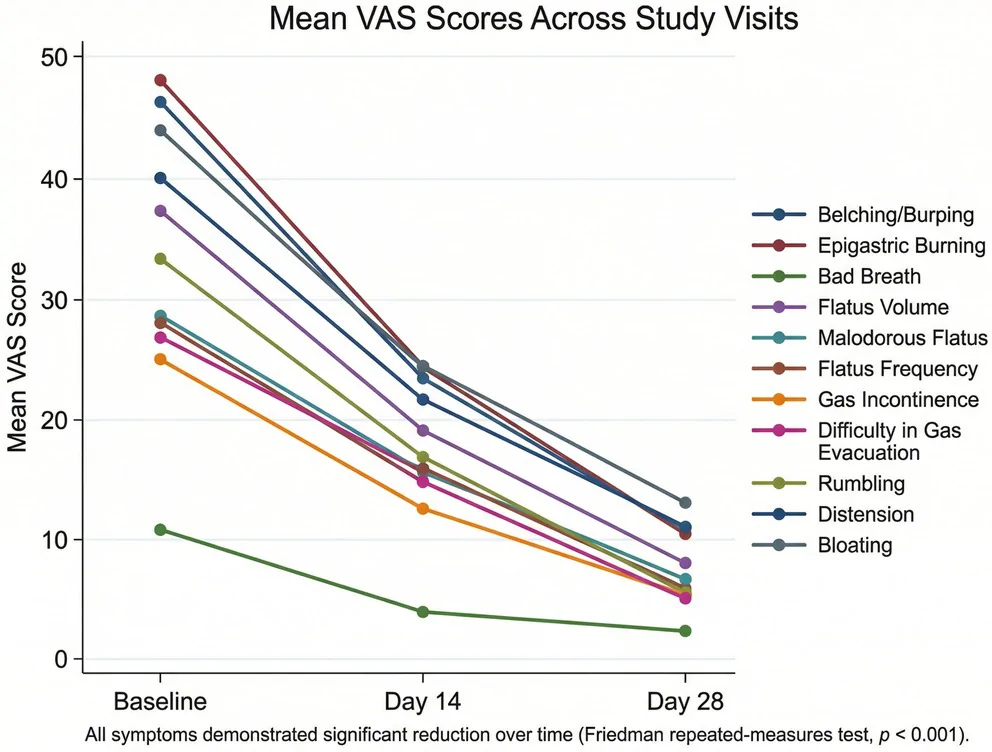
Mean VAS scores across study visits.

#### 3.3.2 Lower Gastrointestinal Symptoms

Excessive flatus volume decreased from 37.3 ± 29.9 at baseline to 19.0 ± 21.1 at Day 14 and 8.1 ± 14.3 at Day 28. Malodorous flatus declined from 28.1 ± 28.1 to 15.8 ± 20.3 and 6.6 ± 13.2, while excessive frequency of flatulence reduced from 28.6 ± 27.1 to 15.3 ± 18.3 and 5.9 ± 11.8 over the same interval. Gas incontinence and difficulty in gas evacuation similarly showed progressive reductions, reaching mean scores of 5.6 ± 11.7 and 4.9 ± 12.3 by Day 28, respectively (Figure 3). All lower gastrointestinal symptoms demonstrated statistically significant changes across visits (Friedman test, *p* < 0.001), with significant pairwise reductions on Bonferroni‐adjusted post hoc testing.

#### 3.3.3 Abdominal Sensation–Related Symptoms

Stomach rumbling decreased from 33.4 ± 28.3 at baseline to 17.1 ± 18.5 at Day 14 and 6.5 ± 14.3 at Day 28. Abdominal distension improved from 40.1 ± 30.5 to 22.0 ± 22.5 and 11.5 ± 18.4, while bloating declined from 44.2 ± 29.1 at baseline to 24.1 ± 23.2 at Day 14 and 12.9 ± 18.6 at Day 28 (Figure 3). As with other domains, reductions were statistically significant across time points (Friedman test, *p* < 0.001).

The absolute mean reductions from baseline to Day 28 ranged from 8.4 points for bad breath to 37.5 points for epigastric burning on the 0–100 scale, corresponding to relative reductions of 71%–81% across major gas‐related domains.

### 3.4 Overall Statistical Analysis

Across all 11 symptoms, Friedman repeated‐measures analysis confirmed statistically significant reductions in ordinal severity from baseline through Day 28 (all *p* < 0.001). Bonferroni‐adjusted Wilcoxon post hoc testing demonstrated significant pairwise improvement at each interval. The largest magnitude of change was observed for belching/burping, epigastric burning, bloating, and abdominal distension. Detailed statistical outputs for all 11 symptoms, including Friedman test statistics and Bonferroni‐adjusted post hoc comparisons, are provided in Supplementary Table S1. Clinician‐rated satisfaction was high, with approximately 89% of patients rated as satisfied or very satisfied for dyspepsia management and 90% for chronic flatulence management; the mean recommendation score was 7.6 ± 1.7 on a 0–10 scale (Table S2). Symptomatic improvement was broadly comparable between patients receiving the combination as add‐on therapy (*n* = 142) and those receiving it as PPI replacement (*n* = 17), with no significant between‐group differences on any symptom domain (Table S3). Temporal analysis indicated that 62% of the total symptomatic improvement observed by Day 28 had already occurred by Day 14, with 71%–85% of symptomatic patients showing early response; the proportion achieving ≥ 50% symptom reduction by Day 28 exceeded 80% for most domains (Table S5). Detailed anthropometric and cardiovascular risk characterization of the study cohort is provided in Table S6.

### 3.5 Subgroup Analysis of Global Symptom Improvement

To evaluate the consistency of treatment response across clinically relevant categories, exploratory subgroup analyses were performed using the median change in global symptom score from baseline to Day 28, as described in the Methods.

The overall median reduction in global symptom score was 25.0 units (95% CI: 22.73–27.27). Across subgroups, median improvements were broadly comparable (Figure 4). Median change ranged from 20.45 to 27.27 units in most subgroups. Participants with constipation demonstrated a numerically lower median improvement (16.36 units), although confidence intervals overlapped with the overall estimate.

**FIGURE 4:**
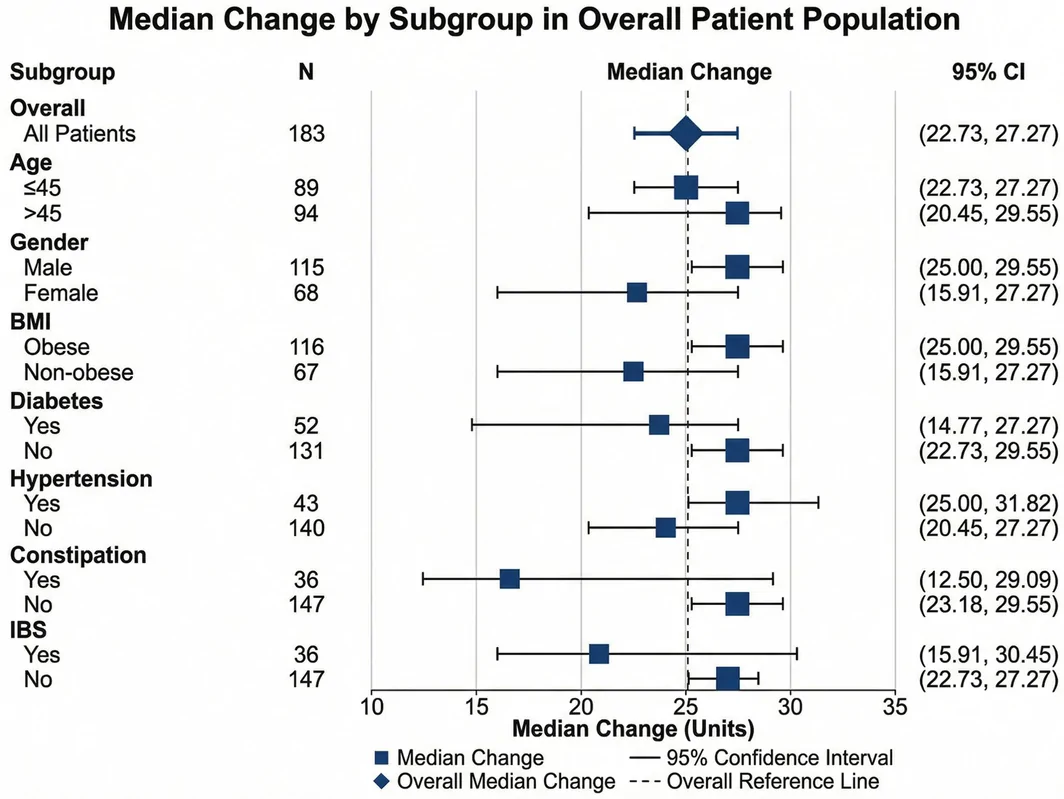
Forest plot of median change in global symptom score (Baseline to Day 28) across predefined subgroups. Squares represent median change within each subgroup; horizontal lines indicate 95% confidence intervals. The dashed vertical line denotes the overall median change. Between‐group comparisons were performed using the Mann–Whitney *U* test. No subgroup differences remained statistically significant after adjustment for multiple comparisons.

Between‐group comparisons were performed using the Mann–Whitney *U* test. Although obese participants showed a numerically greater median reduction compared with non‐obese individuals (27.27 vs. 22.73 units; *p* = 0.027), this difference did not remain statistically significant after adjustment for multiple comparisons. No other subgroup comparisons reached statistical significance (all *p* > 0.05).

Overall, symptomatic improvement was consistent across demographic and clinical subgroups, suggesting that the therapeutic response was not materially influenced by age, sex, metabolic comorbidities, or functional gastrointestinal overlap conditions. Expanded per‐symptom subgroup analyses for diabetes mellitus, hypertension, and obesity, including within‐group Wilcoxon signed‐rank testing, are presented in Table S4.

### 3.6 Treatment Adherence and Safety

Self‐reported adherence data were available for 170 of 183 participants at Day 14 and 171 at Day 28. At Day 14, 148 (87.1%) reported taking the medication as prescribed, 20 (11.8%) missed a few doses, and 2 (1.2%) missed more than eight doses. At Day 28, 143 (83.6%) reported full adherence, 23 (13.5%) missed a few doses, 3 (1.8%) missed more than eight doses, and 2 (1.2%) took the medication on an as‐needed basis only. Detailed adherence and safety data are provided in Table S7.

Safety was assessed using a single patient‐reported item asking whether participants noticed any unusual or unexpected symptoms or changes in health since starting therapy. An affirmative response was reported by 1.8% of participants at Day 14 and 3.0% at Day 28. The nature, severity, and relatedness of these reported changes were not further characterized in the data collection instrument. No serious clinical events were reported during follow‐up, and no treatment discontinuations were documented.

## 4 Discussion

In this prospective, multicenter, real‐world study of patients with persistent dyspeptic symptoms despite prior proton pump inhibitor therapy, treatment with a fixed‐dose combination of simethicone and activated charcoal was associated with consistent and statistically significant reductions in symptom severity across 11 upper and lower gastrointestinal domains over a 28‐day period. Improvement was evident as early as Day 14 and was sustained or further enhanced by Day 28. The magnitude of change was most pronounced for belching, epigastric burning, bloating, and abdominal distension, although meaningful reductions were observed across all evaluated symptoms.

Importantly, symptomatic improvement appeared broadly consistent across demographic and clinical subgroups, including age, sex, obesity status, and common metabolic or functional gastrointestinal comorbidities. After adjustment for multiple comparisons, no subgroup demonstrated a statistically significant differential response, suggesting that the observed benefit was not materially modified by baseline metabolic or functional overlap conditions. Collectively, these findings indicate a reproducible symptomatic signal in a real‐world population characterized by gas‐predominant dyspeptic complaints refractory to prior acid‐suppressive therapy.

Beyond statistical significance, the clinical relevance of the observed improvements merits consideration. No validated minimal clinically important difference (MCID) has been established for the modified instrument used in this study. However, for analogous 0–100 VAS instruments in functional gastrointestinal research, MCID thresholds of 10–15 points are generally accepted. The absolute reductions observed across major symptom domains in this study (25–37 points) substantially exceeded this benchmark, suggesting clinically meaningful improvement. Nevertheless, in the absence of a comparator arm, the proportion of this improvement attributable to the intervention versus placebo response or natural symptom fluctuation cannot be determined.

Functional dyspepsia remains a heterogeneous and frequently refractory condition in routine clinical practice [4]. Although PPIs are commonly prescribed and may provide benefit in acid‐mediated or overlapping gastroesophageal reflux symptoms [13], a substantial proportion of patients continue to experience persistent complaints [26]. In many such individuals, symptoms of bloating, belching, postprandial fullness, abdominal distension, and excessive flatus predominate. These manifestations likely reflect mechanisms beyond acid secretion, including impaired gastric accommodation, altered gut motility, visceral hypersensitivity, and increased intraluminal gas or abnormal gas handling [17]. Notably, placebo response rates in functional dyspepsia trials are high, with meta‐analyses reporting pooled rates of approximately 39%–44% for symptom improvement [27, 28]. While the absence of a placebo arm in the present study precludes direct estimation of net treatment effect, the consistency of improvement across 11 independent symptom domains and two time points provides some reassurance that the findings are not solely attributable to a nonspecific response.

The present study specifically evaluated patients who remained symptomatic despite prior PPI therapy—a clinically relevant subgroup in whom further acid suppression yields diminishing returns. Current guidelines, including the AGA clinical practice update on PPI deprescribing, explicitly identify functional dyspepsia with no sustained response to PPI therapy as an indication that does not support long‐term PPI use [29]. The observed reductions in symptom severity with a luminal, gas‐targeted intervention suggest that addressing intraluminal gas may not only complement residual acid‐suppressive therapy but also potentially facilitate appropriate PPI step‐down or discontinuation in selected patients—an important consideration given the growing emphasis on PPI stewardship [29, 30].

Previous randomized controlled trials have demonstrated the efficacy of simethicone‐based therapies in functional dyspepsia. A simethicone–activated charcoal combination was superior to placebo for overall symptom reduction in two European primary care trials [25, 31], while simethicone monotherapy proved superior to both placebo and cisapride in separate randomized studies [32, 33]. The present observational study complements these controlled data by extending findings to a clinically heterogeneous Indian population with persistent symptoms despite prior PPI therapy—a subgroup often underrepresented in randomized trials. Moreover, prior controlled trials evaluated different formulations containing magnesium oxide (Carbosymag) in European primary care populations and did not specifically target patients with PPI‐refractory symptoms. The present study therefore extends the evidence base to a distinct clinical context—PPI‐refractory gas‐predominant dyspepsia in an Indian population with different dietary and prescribing patterns—while also providing novel data on early onset of response, subgroup consistency, and potential relevance to PPI deprescribing strategies.

It is important to interpret these findings within the context of the study design. As an uncontrolled, single‐arm study, the observed improvements cannot be causally attributed to the intervention. Placebo effects, which account for 39%–44% of therapeutic responses in functional dyspepsia trials [27, 28], regression to the mean in patients enrolled at symptom peaks, natural symptom fluctuation characteristic of functional gastrointestinal disorders, and the Hawthorne effect from study participation itself may all have contributed to the observed reductions. The progressive and sustained improvement between Day 14 and Day 28, rather than a plateau or rebound, and the reproducible reductions across 11 symptom domains are less consistent with simple regression to the mean, but do not exclude these possibilities in the absence of a comparator arm. These findings should therefore be regarded as hypothesis‐generating real‐world evidence rather than proof of efficacy, and warrant confirmation in well‐designed placebo‐controlled randomized trials.

Several potential confounders warrant acknowledgment. Concurrent dietary modifications, which are commonly advised alongside pharmacotherapy in Indian gastroenterology practice, were not systematically recorded and may have contributed independently to symptom improvement. Use of rescue medications during the study period was not captured. Use of concomitant mucosal protective agents was not restricted; review of the medication data revealed that sucralfate (*n* = 11) and alginates (*n* = 2) were used concurrently in a small number of patients, representing a potential confounder. Adherence to ongoing PPI therapy in the add‐on subgroup was not independently monitored, and changes in PPI dose or compliance could have influenced outcomes. The comparable symptomatic improvement observed between the add‐on (*n* = 142) and replacement (*n* = 17) subgroups (Table S3) suggests that the benefit was not solely attributable to continued acid suppression, although the markedly unequal group sizes limit this inference. Formal multivariable adjustment for potential confounders was not feasible given the sample size and observational design. These unmeasured confounders, together with the absence of a comparator arm, underscore the need for controlled trials with standardized co‐intervention protocols.

The generalizability of these findings also warrants consideration. Although the nine study sites spanned geographically diverse regions of India—including eastern, northeastern, northern, and southern centres—the traditional Indian diet, characterized by high legume, fiber, and spice intake, is known to contribute to intraluminal gas production and may have amplified both the baseline symptom burden and the potential for improvement with a gas‐targeted intervention. Whether comparable results would be observed in populations with different dietary patterns remains uncertain. Site‐specific prescribing practices, variable thresholds for defining PPI inadequacy, and differences in patient–physician interaction may have introduced heterogeneity across centres. Furthermore, the study population was drawn from outpatient gastroenterology and general practice settings, and findings may not be generalizable to tertiary referral populations with more refractory functional gastrointestinal disorders.

Subgroup analyses using the global symptom score showed broadly comparable improvements across age, sex, obesity, diabetes, hypertension, constipation, and IBS overlap, without evidence of clinically meaningful effect modification after correction for multiple comparisons. This consistency is clinically relevant given that metabolic comorbidities and overlapping functional bowel symptoms are frequently encountered in patients presenting with dyspepsia. The high clinician‐rated satisfaction and recommendation scores (Table S2) and comparable improvement whether the combination was used as add‐on or replacement therapy (Table S3) further support its acceptability and potential relevance to PPI deprescribing.

One practical consideration relevant to the concurrent use of simethicone–activated charcoal with other medications deserves comment. Activated charcoal has well‐documented adsorptive properties that can reduce the bioavailability of concurrently administered oral drugs [34]. In the present study, the combination was prescribed 1 h after meals, which provides temporal separation from most medications taken with food. However, in clinical practice, clinicians should be mindful of potential interactions, particularly in patients receiving narrow therapeutic index drugs, and should advise appropriate dosing intervals between activated charcoal and other oral medications.

The strengths of this study include its prospective design, multicenter conduct across India, complete follow‐up without attrition, and systematic assessment of symptom severity across a broad range of gas‐related upper and lower gastrointestinal complaints at predefined time points. The use of non‐parametric repeated‐measures testing with post hoc correction strengthens the internal consistency of the statistical analysis. Several limitations merit consideration. First, the study was single‐arm and observational, and therefore cannot exclude placebo response, regression to the mean, or the influence of concurrent therapies and lifestyle modifications. Given the high placebo response rates reported in functional dyspepsia trials [27, 28], this remains an important interpretive caveat. Second, outcomes were based on patient‐reported symptom severity and were not supported by objective physiologic measures such as gastric emptying studies, accommodation assessment, or direct quantification of intestinal gas. Third, no formal adverse event reporting methodology (including MedDRA coding, causality assessment, or severity grading) was employed; safety was assessed using a single patient‐reported item without characterization of event type, severity, duration, or relatedness, and no biochemical monitoring (including renal and hepatic function tests) was performed. While this is consistent with the non‐systemic, non‐absorbed pharmacological profile of both agents and their established safety record, it limits the comprehensiveness and interpretability of the safety conclusions. Fourth, the symptom domains, while selected based on the Intestinal Gas Questionnaire framework, were administered in a modified five‐point ordinal format that has not been independently validated; no claim of validity is made based on the original IGQ. Similarly, the global symptom score used for subgroup comparisons is an unvalidated arithmetic composite of ordinal items and should be interpreted with caution. Future confirmatory studies should incorporate fully validated patient‐reported outcome instruments such as the IGQ or the Patient Assessment of Upper Gastrointestinal Disorders–Symptom Severity Index (PAGI‐SYM). Additional limitations include physician‐based rather than Rome IV‐confirmed diagnosis of functional dyspepsia, absence of *H. pylori* testing, markedly unequal add‐on versus replacement group sizes (142 vs. 17), short follow‐up duration, and absence of systematic PPI dose documentation.

## 5 Conclusion

In this multicenter, prospective, real‐world study of Indian adults with persistent gas‐predominant dyspeptic symptoms despite prior acid‐suppressive therapy, treatment with a fixed‐dose combination of simethicone (80 mg) and activated charcoal (250 mg) three times daily for 28 days was associated with significant and consistent reductions in symptom severity across all 11 evaluated gastrointestinal domains. Improvement was evident by Day 14 and was sustained through Day 28, with broadly comparable responses across demographic and clinical subgroups including patients with diabetes, hypertension, obesity, and overlapping functional bowel disorders. The combination was well tolerated, with high treatment adherence and no serious clinical events reported. These findings support the clinical rationale for a luminal, gas‐targeted therapeutic approach as a complement to, or in selected patients as an alternative to, continued acid‐suppressive therapy in gas‐predominant dyspepsia. Given the single‐arm observational design, these hypothesis‐generating findings warrant confirmation in well‐designed, placebo‐controlled randomized trials to establish treatment effect independent of placebo response, regression to the mean, and natural symptom variability.

## Funding

This study was funded by Indchemie Health Specialities Pvt. Ltd. The funder was involved in study design, data collection, and data management through its contract research partner (MEDEVA). The decision to submit the manuscript and the interpretation of results were the responsibility of the authors.

## Ethics Statement

The study was approved by the Royal Pune Independent Ethics Committee, Pune, India (IEC no. RPIEC090524; registration no. ECR/45/Indt/MH/2013/RR‐19; Protocol id: MED/INDI/2024/SIMAC). The study was conducted in accordance with the Declaration of Helsinki and the International Council for Harmonisation Good Clinical Practice (ICH‐GCP) guidelines.

## Consent

Written informed consent was obtained from all participants prior to enrollment.

## Conflicts of Interest

Ashish Kumar reports relationships with Roche, Hetero, Viatris, La Renon, Torrent, and Sun Pharma, including receipt of speaking and lecture fees as well as travel reimbursement. Rahul Pathak and Shruti Pal are employees of Indchemie Health Specialities Pvt. Ltd. The other authors declare no conflicts of interest.

## Supporting information


**Table S1:** Detailed statistical analysis of symptom severity changes.
**Table S2:** Clinician‐rated satisfaction and recommendation.
**Table S2a:** Overall satisfaction with management of dyspepsia (*n* = 180).
**Table S2b:** Overall satisfaction with management of chronic flatulence (*n* = 181).
**Table S2c:** Likelihood of recommendation (*n* = 149).
**Table S3:** Comparison of symptomatic improvement: add‐on versus replacement therapy.
**Table S4:** Expanded subgroup analyses: per‐symptom improvement in patients with diabetes, hypertension, and obesity.
**Table S4a:** Symptom improvement in patients with diabetes mellitus (*n* = 52).
**Table S4b:** Symptom improvement in patients with hypertension (*n* = 43).
**Table S4c:** Symptom improvement in patients with obesity (BMI ≥ 25 kg/m^2^; *n* = 112).
**Table S5:** Early responder analysis and time‐course of symptomatic improvement.
**Table S5a:** Response pattern by symptom.
**Table S5b:** Proportion achieving ≥ 50% reduction from baseline.
**Table S6:** Anthropometric and cardiovascular risk characterization.
**Table S6a:** BMI distribution (*n* = 176).
**Table S6b:** Abdominal girth risk categories (*n* = 114).
**Table S6c:** Blood pressure distribution (InSH 2023 classification; *n* = 161).
**Table S7:** Detailed safety and adherence data.
**Table S7a:** Treatment adherence.
**Table S7b:** Unusual or unexpected symptoms.

## Data Availability

The data supporting the findings of this study are available from the corresponding author upon reasonable request.
