# Supplementary material for: A Multicenter, Prospective Real‐World Study of Simethicone–Activated Charcoal in Patients With Persistent Dyspeptic Symptoms Despite Acid Suppression (SIMAC Study)
Source: JGH Open. 2026 Oct 1;10(10):e70475. doi: 10.1002/jgh3.70475 (PMC13628556; doi:10.1002/jgh3.70475)
Supplement: Supplementary file 1 [file JGH3-10-e70475-s001.docx]

**SUPPLEMENTARY APPENDIX**

*A Multicenter, Prospective Real-World Study of Simethicone–Activated Charcoal in Patients with Persistent Dyspeptic Symptoms Despite Acid Suppression (SIMAC Study)*

## Supplementary Table S1. Detailed Statistical Analysis of Symptom Severity Changes

Friedman repeated-measures test across three time points (Baseline, Day 14, Day 28) with Bonferroni-adjusted Wilcoxon signed-rank post-hoc pairwise comparisons for each of eleven gastrointestinal symptom domains (N = 183). Values represent mean ± SD. Δ indicates mean difference between time points. All adjusted p-values are Bonferroni-corrected for three comparisons.

| **Symptom** | **Baseline** | **Day 14** | **Day 28** | **χ2** | **p** | **Δ B–D14** | **Adj. p** | **Δ B–D28** | **Adj. p** | **Δ D14–D28** | **Adj. p** |
| --- | --- | --- | --- | --- | --- | --- | --- | --- | --- | --- | --- |
| Belching/burping | 46.3 ± 26.4 | 23.9 ± 20.9 | 10.9 ± 19.5 | 243.3 | <0.001 | 22.4 | <0.001 | 35.3 | <0.001 | 13.0 | <0.001 |
| Epigastric burning | 48.1 ± 27.3 | 24.8 ± 22.2 | 10.7 ± 17.9 | 241.7 | <0.001 | 23.4 | <0.001 | 37.5 | <0.001 | 14.1 | <0.001 |
| Bad breath | 10.8 ± 20.0 | 4.1 ± 10.5 | 2.4 ± 8.7 | 74.0 | <0.001 | 6.7 | <0.001 | 8.4 | <0.001 | 1.7 | 0.012 |
| Excessive flatus volume | 37.3 ± 29.9 | 19.0 ± 21.1 | 8.1 ± 14.3 | 216.5 | <0.001 | 18.3 | <0.001 | 29.2 | <0.001 | 10.9 | <0.001 |
| Malodorous flatus | 28.1 ± 28.1 | 16.0 ± 20.4 | 6.4 ± 12.8 | 162.5 | <0.001 | 12.1 | <0.001 | 21.7 | <0.001 | 9.6 | <0.001 |
| Excessive flatulence frequency | 28.6 ± 27.1 | 15.3 ± 18.3 | 5.7 ± 11.7 | 164.1 | <0.001 | 13.3 | <0.001 | 22.9 | <0.001 | 9.5 | <0.001 |
| Gas incontinence | 25.0 ± 26.5 | 12.6 ± 18.2 | 5.6 ± 11.7 | 155.4 | <0.001 | 12.4 | <0.001 | 19.4 | <0.001 | 7.0 | <0.001 |
| Difficulty in gas evacuation | 27.1 ± 23.9 | 14.2 ± 16.7 | 4.9 ± 12.3 | 182.8 | <0.001 | 12.9 | <0.001 | 22.2 | <0.001 | 9.3 | <0.001 |
| Stomach rumbling | 33.4 ± 28.3 | 17.1 ± 18.5 | 6.5 ± 14.3 | 200.8 | <0.001 | 16.3 | <0.001 | 26.9 | <0.001 | 10.6 | <0.001 |
| Abdominal distension | 40.1 ± 30.5 | 22.0 ± 22.5 | 11.5 ± 18.4 | 183.3 | <0.001 | 18.1 | <0.001 | 28.6 | <0.001 | 10.5 | <0.001 |
| Bloating | 44.2 ± 29.1 | 24.1 ± 23.2 | 12.9 ± 18.6 | 204.5 | <0.001 | 20.1 | <0.001 | 31.3 | <0.001 | 11.2 | <0.001 |

## Supplementary Table S2. Clinician-Rated Satisfaction and Recommendation

Clinician-rated treatment satisfaction was assessed at Day 28 using a five-point Likert scale for two domains: management of dyspepsia and management of chronic flatulence. Likelihood of recommendation was assessed using a 0–10 Numeric Rating Scale. Satisfaction data were available for 180 of 183 participants (dyspepsia domain) and 181 of 183 (flatulence domain). Recommendation scores were available for 149 participants.

**Table S2a. Overall Satisfaction with Management of Dyspepsia (n = 180)**

| **Response** | **n** | **%** |
| --- | --- | --- |
| Very Satisfied | 95 | 52.8 |
| Satisfied | 66 | 36.7 |
| Neutral | 9 | 5.0 |
| Dissatisfied | 8 | 4.4 |
| Very Dissatisfied | 2 | 1.1 |

*Satisfied or Very Satisfied combined: 161 (89.4%)*

**Table S2b. Overall Satisfaction with Management of Chronic Flatulence (n = 181)**

| **Response** | **n** | **%** |
| --- | --- | --- |
| Very Satisfied | 99 | 54.7 |
| Satisfied | 64 | 35.4 |
| Neutral | 7 | 3.9 |
| Dissatisfied | 7 | 3.9 |
| Very Dissatisfied | 4 | 2.2 |

*Satisfied or Very Satisfied combined: 163 (90.1%)*

**Table S2c. Likelihood of Recommendation (n = 149)**

Mean recommendation score: 7.6 ± 1.7 on a 0–10 scale. Median: 8.0 (IQR: 7.0–9.0). The distribution was left-skewed, with 40.3% of clinicians rating a score of 9, 23.5% rating 8, and 16.1% rating 7. Based on Net Promoter Score classification, 40.3% were promoters (score 9–10), 39.6% were passives (score 7–8), and 20.1% were detractors (score 0–6), yielding a Net Promoter Score of +20.1.

*Note: Satisfaction was assessed using a clinician-rated Likert scale rather than a validated patient-reported outcome instrument. Recommendation data were available for a subset of participants (149 of 183); the denominator difference is attributable to incomplete data capture at some sites. These limitations should be considered when interpreting these findings.*

## Supplementary Table S3. Comparison of Symptomatic Improvement: Add-on Versus Replacement Therapy

Simethicone–activated charcoal was prescribed as add-on therapy to ongoing acid suppression in 142 patients and as a replacement for prior PPI therapy in 17 patients. Treatment strategy data were unavailable for 24 participants. Between-group differences in mean VAS change from baseline to Day 28 were compared using the Mann–Whitney U test. The global symptom score (mean of all 11 VAS domains) showed no statistically significant difference between groups (median change 25.0 vs 25.0; p = 0.484).

| **Symptom** | **Add-on (n = 142) Mean Δ ± SD** | **Replacement (n = 17) Mean Δ ± SD** | **p-value** |
| --- | --- | --- | --- |
| Stomach rumbling | 27.8 ± 25.4 | 32.4 ± 23.0 | 0.436 |
| Difficulty in gas evacuation | 23.9 ± 22.8 | 23.5 ± 18.7 | 0.998 |
| Gas incontinence | 21.0 ± 22.4 | 16.2 ± 19.6 | 0.454 |
| Excessive flatus volume | 30.0 ± 23.5 | 33.8 ± 30.5 | 0.439 |
| Malodorous flatus | 22.4 ± 23.4 | 17.6 ± 19.3 | 0.470 |
| Excessive flatulence frequency | 24.2 ± 24.6 | 25.0 ± 25.0 | 0.877 |
| Belching/burping | 34.8 ± 22.3 | 32.4 ± 21.2 | 0.616 |
| Epigastric burning | 37.2 ± 25.4 | 47.1 ± 26.3 | 0.256 |
| Abdominal distension | 25.9 ± 26.3 | 33.8 ± 24.9 | 0.185 |
| Bloating | 29.3 ± 25.1 | 35.3 ± 25.1 | 0.339 |
| Bad breath | 6.8 ± 14.4 | 7.4 ± 14.7 | 0.977 |

*Interpretation: No statistically significant differences were observed between add-on and replacement strategies for any individual symptom domain. The small replacement subgroup (n = 17) limits statistical power and precludes definitive conclusions. However, the broadly comparable improvements in both groups suggest that the combination may have clinical utility in both treatment contexts. These findings should be interpreted as hypothesis-generating and require confirmation in adequately powered comparative studies.*

## Supplementary Table S4. Expanded Subgroup Analyses: Per-Symptom Improvement in Patients with Diabetes, Hypertension, and Obesity

Within-group symptom changes from baseline to Day 28 were assessed using the Wilcoxon signed-rank test for each subgroup. Between-group comparisons of global symptom score change were assessed using the Mann–Whitney U test. Comorbidity data were available for 130 of 183 patients; subgroup denominators reflect available data.

**Table S4a. Symptom Improvement in Patients with Diabetes Mellitus (n = 52)**

| **Symptom** | **Baseline (Mean ± SD)** | **Day 28 (Mean ± SD)** | **Mean Δ** | **p-value** |
| --- | --- | --- | --- | --- |
| Belching/burping | 41.4 ± 24.6 | 12.2 ± 19.4 | 29.2 | <0.001 |
| Epigastric burning | 45.5 ± 28.2 | 13.8 ± 19.3 | 31.6 | <0.001 |
| Bad breath | 8.4 ± 19.3 | 1.8 ± 7.9 | 6.6 | 0.003 |
| Excessive flatus volume | 38.8 ± 33.0 | 10.5 ± 17.2 | 28.3 | <0.001 |
| Malodorous flatus | 31.0 ± 32.4 | 8.4 ± 14.6 | 22.6 | <0.001 |
| Excessive flatulence frequency | 24.7 ± 26.6 | 7.0 ± 14.0 | 17.7 | <0.001 |
| Gas incontinence | 30.1 ± 28.9 | 7.9 ± 14.4 | 22.2 | <0.001 |
| Difficulty in gas evacuation | 30.0 ± 25.0 | 8.6 ± 17.6 | 21.4 | <0.001 |
| Stomach rumbling | 27.6 ± 30.4 | 8.1 ± 17.5 | 19.6 | <0.001 |
| Abdominal distension | 38.2 ± 28.2 | 13.9 ± 21.7 | 24.3 | <0.001 |
| Bloating | 44.4 ± 29.6 | 15.8 ± 20.9 | 28.6 | <0.001 |

*Global symptom score change: median 23.86 (DM) vs 29.55 (non-DM); Mann–Whitney U p = 0.016. Note: this difference did not survive Bonferroni correction for multiple subgroup comparisons.*

**Table S4b. Symptom Improvement in Patients with Hypertension (n = 43)**

| **Symptom** | **Baseline (Mean ± SD)** | **Day 28 (Mean ± SD)** | **Mean Δ** | **p-value** |
| --- | --- | --- | --- | --- |
| Belching/burping | 47.7 ± 29.8 | 12.4 ± 15.7 | 35.2 | <0.001 |
| Epigastric burning | 55.8 ± 31.3 | 12.2 ± 17.6 | 43.6 | <0.001 |
| Bad breath | 9.3 ± 16.4 | 0.6 ± 3.8 | 8.7 | 0.001 |
| Excessive flatus volume | 41.3 ± 34.5 | 8.7 ± 15.3 | 32.6 | <0.001 |
| Malodorous flatus | 33.6 ± 32.5 | 7.6 ± 12.8 | 26.0 | <0.001 |
| Excessive flatulence frequency | 30.3 ± 31.8 | 6.4 ± 13.5 | 24.0 | <0.001 |
| Gas incontinence | 31.5 ± 31.1 | 7.0 ± 12.6 | 24.5 | <0.001 |
| Difficulty in gas evacuation | 29.1 ± 27.8 | 6.4 ± 12.3 | 22.7 | <0.001 |
| Stomach rumbling | 41.6 ± 35.7 | 12.2 ± 19.2 | 29.4 | <0.001 |
| Abdominal distension | 46.3 ± 30.3 | 11.6 ± 16.7 | 34.7 | <0.001 |
| Bloating | 53.5 ± 31.1 | 13.4 ± 16.7 | 40.1 | <0.001 |

*Global symptom score change: median 27.27 (HTN) vs 25.00 (non-HTN); Mann–Whitney U p = 0.215 (not significant).*

**Table S4c. Symptom Improvement in Patients with Obesity (BMI ≥25 kg/m²; n = 112)**

| **Symptom** | **Baseline (Mean ± SD)** | **Day 28 (Mean ± SD)** | **Mean Δ** | **p-value** |
| --- | --- | --- | --- | --- |
| Belching/burping | 48.3 ± 25.6 | 11.2 ± 19.1 | 37.1 | <0.001 |
| Epigastric burning | 51.0 ± 26.8 | 12.2 ± 18.9 | 38.9 | <0.001 |
| Bad breath | 12.8 ± 21.4 | 3.0 ± 9.7 | 9.8 | <0.001 |
| Excessive flatus volume | 41.1 ± 29.6 | 9.4 ± 15.5 | 31.7 | <0.001 |
| Malodorous flatus | 34.1 ± 28.5 | 7.7 ± 13.6 | 26.4 | <0.001 |
| Excessive flatulence frequency | 30.1 ± 27.0 | 6.6 ± 12.1 | 23.5 | <0.001 |
| Gas incontinence | 29.1 ± 25.8 | 6.4 ± 12.1 | 22.7 | <0.001 |
| Difficulty in gas evacuation | 31.7 ± 23.2 | 6.3 ± 14.2 | 25.3 | <0.001 |
| Stomach rumbling | 36.9 ± 28.6 | 7.9 ± 14.3 | 28.9 | <0.001 |
| Abdominal distension | 42.6 ± 29.1 | 13.0 ± 19.1 | 29.6 | <0.001 |
| Bloating | 45.7 ± 28.1 | 15.2 ± 18.7 | 30.5 | <0.001 |

*Global symptom score change: median 27.27 (obese) vs 22.73 (non-obese); Mann–Whitney U p = 0.046. Note: this difference did not survive Bonferroni correction for multiple subgroup comparisons.*

## Supplementary Table S5. Early Responder Analysis and Time-Course of Symptomatic Improvement

Temporal response patterns were characterized among patients who reported each symptom at baseline (VAS score > 0). Patients were classified as early responders (any improvement by Day 14), late responders (no improvement by Day 14 but improved by Day 28), or non-responders (no improvement by Day 28). The global symptom score trajectory showed that 62% of the total improvement from baseline to Day 28 had already occurred by Day 14, indicating a predominantly early onset of symptomatic benefit.

**Table S5a. Response Pattern by Symptom**

| **Symptom** | **n with symptom** | **Early responder, n (%)** | **Late responder, n (%)** | **Non-responder, n (%)** |
| --- | --- | --- | --- | --- |
| Stomach rumbling | 125 | 104 (83.2) | 16 (12.8) | 6 (4.8) |
| Difficulty in gas evacuation | 119 | 87 (73.1) | 25 (21.0) | 7 (5.9) |
| Gas incontinence | 101 | 78 (77.2) | 15 (14.9) | 9 (8.9) |
| Excessive flatus volume | 133 | 113 (85.0) | 12 (9.0) | 8 (6.0) |
| Malodorous flatus | 110 | 81 (73.6) | 24 (21.8) | 7 (6.4) |
| Excessive flatulence frequency | 111 | 82 (73.9) | 22 (19.8) | 8 (7.2) |
| Belching/burping | 160 | 123 (76.9) | 27 (16.9) | 12 (7.5) |
| Epigastric burning | 159 | 125 (78.6) | 22 (13.8) | 13 (8.2) |
| Abdominal distension | 138 | 106 (76.8) | 12 (8.7) | 21 (15.2) |
| Bloating | 154 | 110 (71.4) | 25 (16.2) | 19 (12.3) |
| Bad breath | 53 | 43 (81.1) | 7 (13.2) | 4 (7.5) |

*Early responder: any improvement from baseline by Day 14. Late responder: no improvement by Day 14 but improved by Day 28. Non-responder: no improvement by Day 28.*

**Table S5b. Proportion Achieving ≥50% Reduction from Baseline**

| **Symptom** | **n with symptom** | **≥50% by Day 14, n (%)** | **≥50% by Day 28, n (%)** |
| --- | --- | --- | --- |
| Stomach rumbling | 125 | 73 (58.4) | 118 (94.4) |
| Difficulty in gas evacuation | 119 | 68 (57.1) | 110 (92.4) |
| Gas incontinence | 101 | 62 (61.4) | 92 (91.1) |
| Excessive flatus volume | 133 | 83 (62.4) | 120 (90.2) |
| Malodorous flatus | 110 | 62 (56.4) | 97 (88.2) |
| Excessive flatulence frequency | 111 | 68 (61.3) | 102 (91.9) |
| Belching/burping | 160 | 104 (65.0) | 142 (88.8) |
| Epigastric burning | 159 | 105 (66.0) | 141 (88.7) |
| Abdominal distension | 138 | 80 (58.0) | 110 (79.7) |
| Bloating | 154 | 92 (59.7) | 126 (81.8) |
| Bad breath | 53 | 38 (71.7) | 49 (92.5) |

**Global Symptom Score Trajectory**

The mean global symptom score was 33.6 ± 14.6 at baseline, 17.5 ± 11.3 at Day 14, and 7.8 ± 8.9 at Day 28. The mean reduction from baseline to Day 14 was 16.0 units, representing 62% of the total 25.8-unit improvement observed by Day 28. The remaining 38% of improvement (9.8 units) occurred between Day 14 and Day 28. This indicates that a substantial majority of the therapeutic benefit was achieved within the first two weeks of treatment.

## Supplementary Table S6. Anthropometric and Cardiovascular Risk Characterization

Anthropometric and cardiovascular risk parameters were assessed at baseline. BMI was calculated from recorded height and weight and classified according to Indian consensus criteria (InSH 2023). Abdominal girth was available for 114 of 183 participants.

**Table S6a. BMI Distribution (n = 176)**

| **Category** | **n** | **%** |
| --- | --- | --- |
| Underweight (<18.5) | 7 | 4.0 |
| Healthy (18.5–22.99) | 25 | 14.2 |
| Overweight (23–24.9) | 32 | 18.2 |
| Obesity Class I (25–29.9) | 83 | 47.2 |
| Obesity Class II (≥30) | 29 | 16.5 |

*Mean BMI: 26.4 ± 5.0 kg/m². BMI data were unavailable for 7 participants.*

**Table S6b. Abdominal Girth Risk Categories (n = 114)**

| **Category** | **n** | **%** |
| --- | --- | --- |
| Low Risk (<31.5 inches) | 12 | 10.5 |
| Moderate Risk (31.5–35.5 inches) | 34 | 29.8 |
| High Risk (35.6–38.9 inches) | 36 | 31.6 |
| Very High Risk (≥39 inches) | 32 | 28.1 |

*Mean abdominal girth: 40.7 ± 15.7 inches. Median: 36.0 inches (IQR: 34.0–39.9). Abdominal girth data were available for 114 of 183 participants.*

**Table S6c. Blood Pressure Distribution (InSH 2023 Classification; n = 161)**

| **Category** | **n** | **%** |
| --- | --- | --- |
| Optimal (SBP <130 and DBP <85) | 76 | 47.2 |
| High Normal (SBP 130–139 / DBP 85–89) | 58 | 36.0 |
| Grade 1 Hypertension (SBP 140–159 / DBP 90–99) | 21 | 13.0 |
| Grade 2 Hypertension (SBP ≥160 / DBP ≥100) | 6 | 3.7 |

## Supplementary Table S7. Detailed Safety and Adherence Data

**Table S7a. Treatment Adherence**

| **Response** | **Day 0–14, n (%)** | **Day 14–28, n (%)** |
| --- | --- | --- |
| Took as prescribed | 148 (87.1) | 143 (83.6) |
| Missed a few doses (≤5–6 times) | 20 (11.8) | 23 (13.5) |
| Missed >8 doses | 2 (1.2) | 3 (1.8) |
| Took as needed only | — | 2 (1.2) |
| Total with data | 170 | 171 |
| Missing | 13 | 12 |

**Table S7b. Unusual or Unexpected Symptoms**

| **Response** | **Day 14, n (%)** | **Day 28, n (%)** |
| --- | --- | --- |
| No | 172 (98.3) | 171 (97.2) |
| Yes | 3 (1.7) | 5 (2.8) |
| Total with data | 175 | 176 |
| Missing | 8 | 7 |

*Among the three patients reporting unusual symptoms at Day 14, one provided a description: bloated stomach and uneasiness. The same patient reported identical symptoms at Day 28. No further characterization (severity, duration, or relatedness to study medication) was captured by the data collection instrument. No serious clinical events, hospitalizations, or treatment discontinuations were reported.*
